# Using co-occurrence network structure to extract synonymous gene and protein names from MEDLINE abstracts

**DOI:** 10.1186/1471-2105-6-103

**Published:** 2005-04-22

**Authors:** AM Cohen, WR Hersh, C Dubay, K Spackman

**Affiliations:** 1 Department of Medical Informatics and Clinical Epidemiology School of Medicine Oregon Health & Science University 3181 S.W. Sam Jackson Park Road, Mail Code: BICC Portland, Oregon, 97239-3098, USA

## Abstract

**Background:**

Text-mining can assist biomedical researchers in reducing information overload by extracting useful knowledge from large collections of text. We developed a novel text-mining method based on analyzing the network structure created by symbol co-occurrences as a way to extend the capabilities of knowledge extraction. The method was applied to the task of automatic gene and protein name synonym extraction.

**Results:**

Performance was measured on a test set consisting of about 50,000 abstracts from one year of MEDLINE. Synonyms retrieved from curated genomics databases were used as a gold standard. The system obtained a maximum F-score of 22.21% (23.18% precision and 21.36% recall), with high efficiency in the use of seed pairs.

**Conclusion:**

The method performs comparably with other studied methods, does not rely on sophisticated named-entity recognition, and requires little initial seed knowledge.

## Background

The volume of published biomedical research, and therefore the underlying biomedical knowledge base, continues to grow. The MEDLINE 2004 database is currently growing at the rate of about 500,000 new citations each year [1]. With such growth, it is challenging to keep up-to-date with all of the new discoveries and theories even within one's own field of research. Methods must be established to aid biomedical researchers in making better use of the existing published research and helping them put new discoveries into practical use [2].

Text mining and knowledge extraction are ways to aid biomedical researchers in identifying important connections within information in the biomedical knowledge base. A subset of natural language processing (NLP), text mining and knowledge extraction concentrate on solving a specific problem in a specific domain identified *a priori*. For example, literature searching may be improved by identifying all of the names and symbols used in the literature to identify a particular gene [3], or potential new treatments for migraine may be determined by looking for pharmacological substances that regulate biological processes associated with migraine [4,5].

Similar to acronym and abbreviation extraction, which has been studied by several groups [6-8], the problem of gene and protein name synonymy is one that can be addressed with the aid of text mining. Many genes and proteins have multiple names with several orthographic and lexical variants. Gene names are often not used consistently, and new names continue to be created [9,10]. Many attributes of a gene, such as its phenotypes and polymorphisms, may lead to it being given several names over time. Also, genes may have names that are later retracted when new information becomes available [11].

While databases of gene names exist, they have several limitations. Gene name databases such as FlyBase [12] and Genew [13] are restricted to a single species (fruit flies and humans, respectively). LocusLink includes genes and names for several species, but does not attempt to include all names, symbols, and lexical variations that refer to a gene. The Genew database was created by the Human Genome Organisation for the purpose of establishing an approved set of unique gene names and symbols for every gene in the human genome [14]. However, Genew is focused on creating the set of gene names recommended for use in biomedical writing. It is not intended to be a complete collection of the gene names and symbols actually used in the biomedical literature [15].

Since the gene names and symbols used in a journal article are fixed once published, later correction of improper names does not affect the prior published literature. Therefore, the name space representing a gene can become quite large between the time a gene is first suspected and when it is well studied and has a universally agreed upon name. In addition, gene and protein names overlap. They are often used in place of one another within the literature, with the intended gene or protein being dependent upon context. When conducting a literature review, it is useful to search for both gene and protein names simultaneously [9]. Therefore in this work we make no distinction between names of genes and the names of the proteins for which they encode.

An automatically generated list of synonyms would be a useful aid in searching the biomedical literature. These could then be used to improve the recall of genomics investigators trying to find all known information on a gene or protein, regardless of the name or names used in a specific article, although a decrease in precision may result in cases where some of the symbols are shared by multiple genes. An automatically generated list of name synonyms would also be useful in further work on extracting other genomics information from textual sources [16]. To make efficient use of the available data when mining the biomedical literature for relationships, it is important to recognize differing names for identical concepts and treat these as a single concept [17].

The basic idea of name synonym extraction is to automatically extract synonymous names for a given concept from natural language text. In this case, the goal is to extract the names and symbols referring to an individual gene from MEDLINE abstracts. There is significant prior work in this area, done over the last five years by Yu and Agichtein. Yu [18] first worked on gene name synonym extraction with a system that extracted gene name synonyms based on manually identified patterns in which gene name synonyms commonly occur. Domain experts were used to identify common patterns. Yu et al. estimated the precision of their system to be approximately 71%. Recall measurements were not published.

Yu and Agichtein [3] then worked together to combine several gene and protein name synonym text-mining approaches. Their best single system, a pattern-based system named *Snowball*, was based on Brin's Dual Iterative Pattern Expansion (DIPRE) system for the Web [19], which had previously been adapted for extracting relationships from large text collections [20]. A small set of initially known facts is used to find the patterns in which these facts occur within a large corpus. Then these patterns were used to extract more facts, which in turn were used to find more patterns.

Yu and Agichtein combined four approaches, including *Snowball*, and *GPE*, a system based on labor-intensive manually created patterns and heuristic rules, into a single system, by computing the overall system confidence in each synonym pair. The overall confidence measure for the *Combined *systems was defined as one minus the probability that all of the other systems are incorrect, which is the product of one minus the individual confidences. They found that the *Combined *approach worked better than any individual approach, producing a recall of about 80% with a precision of about 9%.

Automatic gene and protein synonym extraction systems have not been put into general use, perhaps because the current level of performance is inadequate for many purposes. It is therefore important to investigate alternative and complementary approaches. Additionally, since the primary work in this area has been done by a single group of investigators, it is essential that other researchers investigate this problem to verify the reproducibility of the results.

## Results

Running our system on the test collection for 9 iterations took approximately 14.5 hours on a 1.7 GHz Pentium 4 with 512 M of RAM. For rapid prototyping the system was implemented in Python, an interpreted language. It is expected that recoding in a compiled language could substantially reduce the execution time.

The experiment produced two kinds of results: performance measures and error analysis. The performance measures summarize the quality of the extracted information. Error analysis provides insight into the strengths and weaknesses of the approach.

### Performance measures

System performance was measured using the precision, recall, and F-score of the extracted set of synonym pairs, as well the absolute and relative number of correct pairs extracted, cumulative for each iteration. Precision is defined as the number of correct pairs, divided by the number of pairs extracted. Recall is defined as the number of pairs extracted that are also present in the recall gold standard, divided by the number of pairs in the recall gold standard. The F-score is the harmonic mean of precision and recall, defined as 2*precision*recall / (precision + recall) [9].

**Figure 1:**
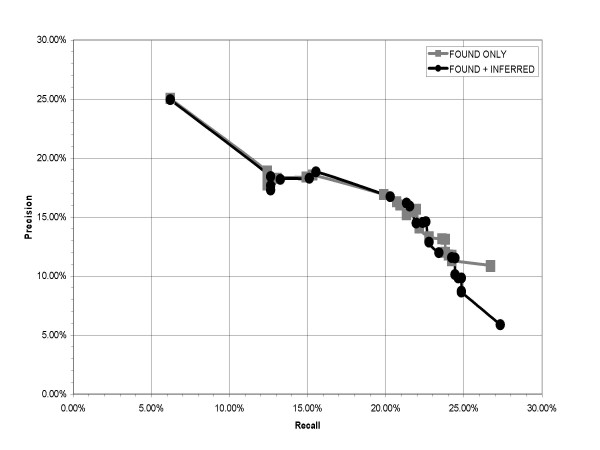
Precision versus recall over all iterations.

Figure 1 shows precision versus recall of the extracted synonym pairs, starting with the first iteration at the left-most point and continuing to the 25th iteration at the right-most point. The graph includes plots of both FOUND pairs (synonym pairs explicitly found in the text by the patterns), as well as FOUND plus INFERRED synonyms (pairs inferred by the graph traversal algorithm). The first iteration achieved a precision of about 25.0%, at a recall of about 6.2%. Precision declines and recall increases practically monotonically over the 24 following iterations to a high recall of about 27.3%, and a precision low of 5.9%.

**Figure 2:**
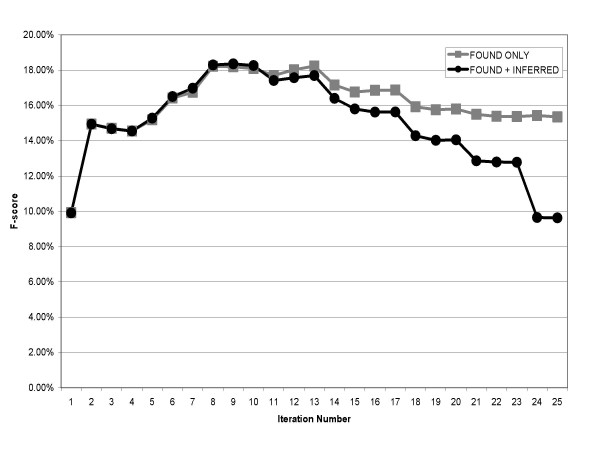
F-score versus iterations.

Figure 2 presents the F-score at each iteration, and again the graph includes plots of both FOUND synonyms as well as FOUND+INFERRED. The maximum F-score of 18.35% for FOUND+INFERRED occurs at iteration 9 (precision 16.18%, recall 21.33%), gradually falling off during subsequent iterations. The use of inference does not greatly impair the algorithm's overall accuracy (as measured by the F-score) until approximately iteration 15.

**Figure 3:**
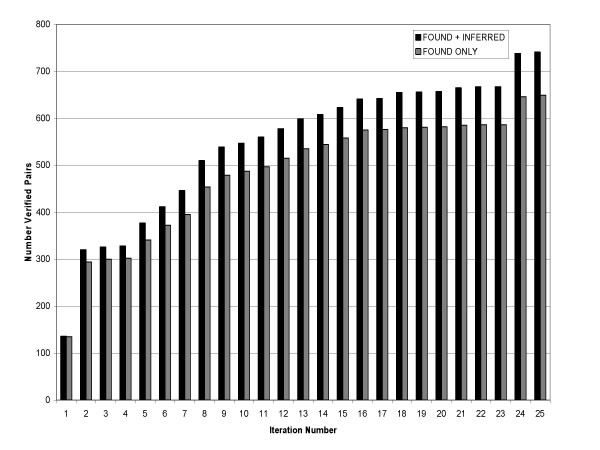
Number verified pairs versus iterations.

The absolute number of correct pairs extracted is presented in Figure 3. Including pairs identified using the inference capability of the network consistently found more pairs than not using the inference capability. At the maximum F-score the system using FOUND+INFERRED synonyms extracted 539 correct synonym pairs, including only the FOUND pairs yielded 479 synonym pairs. The approximately 10% (12.5% at iteration 9) difference in extracted pairs is fairly consistent across all iterations after the initial iteration.

**Figure 4:**
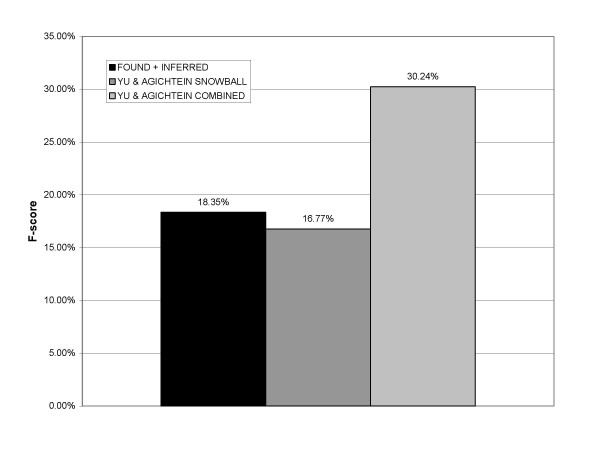
Verified F-score comparison with work of Yu and Agichtein.

Figure 4 compares the results of our system with those of Yu and Agichtein's *Snowball *(their best automated pattern-based approach) and *Combined *(their best overall approach) systems, interpolated from published graphs. The maximum F-score we obtained is comparable with that of *Snowball *(16.77%, precision 52%, recall 10%), but less than that of the *Combined *system (30.24%, precision 62%, recall 20%). The combined system of Yu and Agichtein had superior performance to any single method.

**Figure 5:**
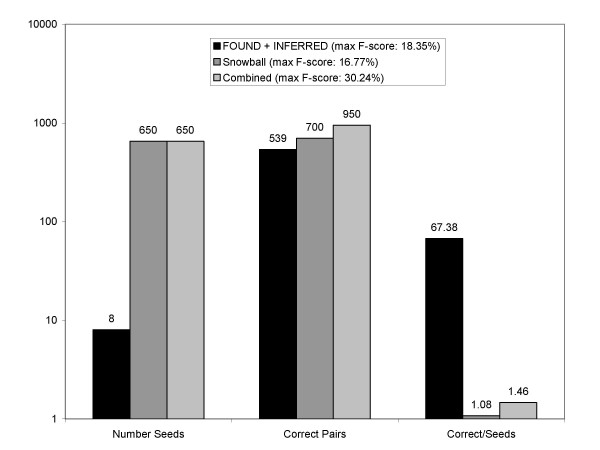
Number seeds, verified pairs extracted, and extraction efficiency.

Another useful measure of system performance is the amount of knowledge extracted per unit of instance knowledge input to the system. This can be interpreted as a measure of how efficiently the algorithm uses the seed data. Figure 5 compares the number of correct extracted pairs to the number of seeds used by our system and those of Yu and Agichtein. Results are shown at the point of maximum F-score in order to provide a consistent comparison. Our system used 8 seed pairs, and 539 correct synonym pairs were extracted. The *Snowball *and *Combined *systems used 650 seed pairs and extracted 700 and 950 correct synonym pairs respectively. The number of correct pairs divided by number of seeds used gives a ratio of 67.38 for our method, with the other systems having much smaller ratios of 1.08 and 1.46 respectively. The *Snowball *and *Combined *systems may not have actually required all 650 seed pairs given as input. However, peak performance of these systems was achieved after only two iterations, implying that the large number of seeds had a substantial influence on the reported results. Further study on the *Snowball *and *Combined *systems is needed is determine how many seed pairs are actually required.

### Error analysis

Two kinds of errors were studied, precision errors and recall errors. Precision errors occurred when the algorithm extracted symbol pairs that were later not verified as synonyms by the precision gold standard data set. These are false positives. Recall errors occurred when the algorithm failed to extract symbol pairs present in the recall gold standard data set. These are false negatives. Errors were studied at the point of maximum F-score, iteration 9.

#### Recall error analysis

Recall errors were categorized into two pre-defined and mutually exclusive categories, *No matching pattern*, and *Pattern not accepted*. The *No matching pattern *error category included all recall errors for which the pattern generation routines failed to identity a pattern that matched the given pair in the abstract text. *Pattern not accepted *errors included those recall errors for which a matching pattern was found, but the matching pattern or patterns were not accepted during the pattern selection optimization step. Using a random sample of 100 false negatives, the majority, 65%, were attributed to the system failing to generate a pattern that matched the recall synonym pair. The remaining 35% of recall errors were due to matching patterns not being accepted by the pattern optimization step.

Some of the recall errors identified as *No matching pattern *may be fixable using a more flexible matching algorithm. In the current system, exact word matching is required of surrounding and intervening words. Small variations in contextual words may have made the algorithm fail to extract a synonym pair that could have been found with more flexible pattern matching. For example, our system treats the patterns "$GENE$($GENE) gene" and "$GENE$/$GENE gene" as completely separate patterns. A more flexible "fuzzy" matching system could allow a pair of gene names followed by the word "gene" to be treated as variants of a single pattern. This approach requires additional tuning to determine how close is "close enough" for a fuzzy match.

Other recall errors identified as *No matching pattern *may be unavoidable in an approach such as ours if the error arises from a synonym pair that only occurs within text pattern not used by any other synonym pair, that is, the text surrounding the synonym pair is unique. This can happen when the synonyms are close together in the text, or when many words separate the synonyms in the text. Because they are long, these patterns will most often be unique. For example, the pair (AHC, NR0B1) is only found in two places in the test collection, in both cases separated by many unique words:

DAX1 encoded by NR0B1, when mutated, is responsible for X-linked adrenal hypoplasia congenita (AHC). [21]

Mutations in DAX1 [dosage-sensitive sex reversal-adrenal hypoplasia congenita (AHC) critical region on the X chromosome gene 1; NR0B1] cause X-linked AHC, a disease characterized by primary adrenal failure in infancy or childhood and reproductive abnormalities later in life. [22]

#### Precision error analysis

Precision errors were categorized by first reviewing a small random sample of 20 errors. From this pilot set of errors, a set of mutually exclusive precision error categories was determined by inspection. The resulting set of six error categories was then applied to an additional random sample of 100 precision errors. The six categories of precision error and the proportions found were:

*(1) Not a gene name *(28%). One or both symbols were not the name of a gene, allele, mutation, or gene family. For example, in the pair (GLN, HBD-2) GLN is an abbreviation for the amino acid glycine.

*(2) Partial gene name *(9%). One or both symbols were part of an incompletely extracted gene name pair. For example in the pair (MAPK, P38), MAPRK1, and MAPK2 are synonyms of P38.

*(3) Biochemically related *(48%). Two different genes that have been reported to interact within the context of a biochemical mechanism, or, names for two distinct genes from the same functionally related family. For example in the pair (IGFI, IGFII) the genes are both members of the family of insulin-like growth factors, and in the pair (BCR, ABL1) the fusion of the BCR and ABL1 genes has been found to be a "recurrent aberration in B cell precursor leukemia cells" [23].

*(4) Unrelated genes *(3%). Two complete gene names but we were unable to establish family or biochemical relationship by reviewing the test data set or MEDLINE. For example in the pair (ARC, CH3) the names are both genes, and were not found to co-occur in the literature. This error is most likely caused by the inference of synonyms from other synonym pairs.

*(5) Mutation variants *(5%). Two mutation names for the same gene but nonspecific for that gene. These are allele or mutation names that are generic and/or only used within a single abstract. For example, the allele A1, or the pair (CYS106ALA, CYS7ALA).

*(6) Correct *(7%). A correct gene synonym pair not included in the gold standard dataset, found later during error analysis by abstract review.

By far, the most commonly occurring error was a pair of gene symbols being chemically or biologically related but distinct, non-synonymous entities. These errors accounted for 48% of the total. The next most common error, occurring 28% of the time, resulted when one or both of the extracted pair of symbols were not a gene or protein name or symbol. The remaining errors were much less common.

#### Incorporation of error analysis into performance results

**Figure 6:**
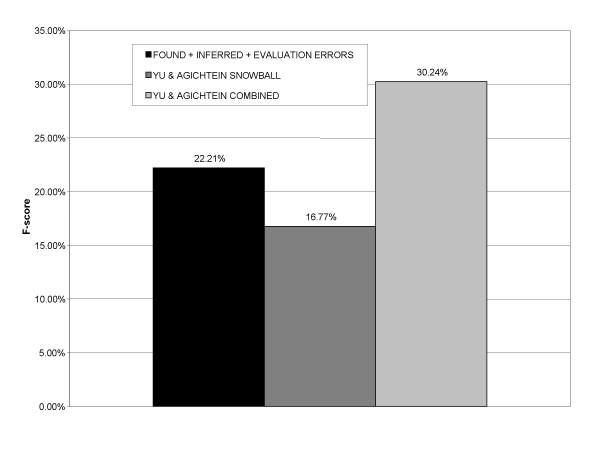
Estimated F-score comparison with work of Yu and Agichtein.

About 7% of precision errors were later determined to be false negatives, that is, the synonym pair was determined by manual inspection of the literature to be correct but was not part of the gold standard data set. Incorporating this proportion of additional correct synonym pairs back into the performance measures previously shown results in an estimated precision of 23.18% and an estimated peak F-score of 22.21%. A comparison of this performance estimate with prior work is shown in Figure 6.

## Discussion

Our results demonstrate that this method compares well to other automated methods of synonym extraction and is a useful general approach to knowledge extraction. The method is highly efficient in its use of seed pairs. This may be an advantage in situations where large numbers of seed pairs are difficult or expensive to collect.

During training, it was determined that using eight initial seed pairs was adequate. It was observed that the performance was largely stable for different initial numbers of seed pairs between 8 and 32. This suggests that an initial "critical mass" of seed pairs was necessary to get the process started. Beyond the critical number, the algorithm automatically found additional common seeds. Including additional common synonym pairs as seeds simply gave as input high confidence pairs that the algorithm could find on its own.

Optimizing the network structure based on the quality metric of the overall network MCC/MNCC (see Methods section) ratio was an effective way to pick the best text patterns for gene synonym pair extraction. Using the symbolic network to support inference of synonym pairs improved both the recall as well as the absolute number of synonym pairs discovered, consistently finding approximately 10% more verified pairs. While there was some loss in precision for these additional pairs, the cost was modest until well past the peak F-score iteration. The inference capability added to its utility as a tool in knowledge discovery, and helped extract additional synonym pairs beyond those found strictly in the text.

One way to improve system performance would be to reduce the very common *Biochemically related *errors by filtering the results to remove known associated gene pairs. There are several on-line databases of gene relationship networks [24,25], and the information in these databases could be used as evidence of the genes being distinct and non-synonymous. While it is unlikely that this filtering could remove all of the false positives from this large source of error, the improvement is likely to be significant.

The relative frequencies of the two types of recall errors present evidence suggesting a general observation about pattern-based text relationship mining systems. Two-thirds of the recall errors were due to the system not having discovered a pattern that matched the non-recalled pair, and only one-third of errors were due to the system having found a matching pattern, rejecting it based on the network metric criteria. The current system used a large number of very specific patterns based on the text surrounding high confidence gene symbol pairs. The *Snowball *system used more flexible patterns, allowing "fuzzy" matching based on the relative importance of word in a pattern. The two different systems performed similarly, which may be due to some inherent limitation of the pattern-based approach to uncovering gene synonym relationships. The textual context of interesting biological relationships may not be specific enough to significantly improve performance. Certainly, more work is needed in this area before drawing strong conclusions.

Since there is no standard test collection for gene symbol synonym extraction research and no absolute gold standard for recall, the recall standard used was an approximation. The method of constructing a recall standard used in this work facilitated comparison with prior work in the field. However, it was by nature a biased sampling method, and does not completely characterize the recall capabilities of current knowledge extraction systems as compared to manual expert review.

The full text test collection previously used by Yu and Agichtein was not publicly available. Major limitations of our study include the lack of a widely available full text test collection of adequate size and the inability to use the same test collection as previous investigators. MEDLINE abstracts were used because they are plentiful and readily available. While prior investigators have stated that full text articles are better sources data for the extraction of gene name synonyms [18], it was encouraging to find that applying our method only to the article abstracts produced comparable results.

The performance of the current system is limited somewhat by the simple orthographic approach used for named-entity recognition (NER). Gene names and symbols were required to be a single string delimited by spaces and other punctuation characters. Not all gene names fit this description, although the gene name pairs extracted for the recall gold standard from SWISSPROT met this requirement. Precision error analysis showed that approximately 28% of precision errors were due to a non-gene or protein symbol being treated as a gene or protein. Another 9% of precision errors were due to an incomplete portion of a gene symbol being identified as a gene symbol. These two categories together represent failure of named entity recognition (NER) and account for 37% of precision errors. Current state-of-the-art F-score performance of biological named-entity recognition is approximately 80% [26]. Using this number as the measure of performance, it can be estimated that the maximum improvement that could be obtained by incorporating a state-of-the-art gene and protein named-entity recognizer into the system would decrease these errors 20%, and increase the precision at the peak F-score to 27%. The actual improvement is likely to be less than the maximum if the NER system makes use of the same contextual information used by the synonym extraction system.

There are many other potential applications of our general approach to mining the biomedical literature. Many inter-entity relationships, such as enhance/inhibit relations between drugs, biological substances, and diseases, and the promoter/suppressor relationships between genes could be modeled as graph structures and appropriate metrics created to measure the relevant network properties. Multiple separate networks can be created simultaneously and then used together during the logical inference step to extend the approach to multiple types of entities and multiple types of relationships between those entities. Further work is necessary to determine whether extracting enhance/inhibit and other functional relations from biomedical text is amenable to our approach. Automatic extraction of complex functional relationships is likely to be more complex than the extraction of synonyms.

Perhaps the most exciting application for the network-based approach is in mining the biomedical literature for hypothesis generation, such as that done manually by Swanson [27], and automatically by others [28,29]. While the Swanson approach is limited to relations between three entities, the network approach can support practically limitless intermediate inferences, limited largely by the confidence in the individual relationships. Future refinements will have to go beyond the simple method used in the current work to determine which relationships are strong enough to support inference. The chain of inference can be modeled as a confidence path with each link reducing the confidence in the entire path by a fraction based on the uncertainty of the relationship.

Having the ability to infer useful hypotheses across several intermediate relationships has the exciting potential to accelerate the rate of medical progress and focus efforts on the most promising prospects. With the biomedical knowledge and the corresponding *bibliome *growing at an exponential rate, the raw material exists for computer assisted hypothesis generation. Further work on text mining and knowledge extraction will be necessary in order to better understand the problems to which it can be most usefully applied, as well as the means to evaluate these systems in order for text mining and knowledge extraction to realize its full potential.

## Conclusion

These results support the conclusion that our method is useful in extracting gene and protein name synonym relationships from biomedical literature abstracts. The current system could be improved by incorporating state-of-the-art NER, and by including additional domain knowledge from richer data sources such as full text articles, and gene network databases which could provide data for negative examples. Use of negative examples could be incorporated into our approach by adding a penalty for extracting negative examples to the genetic optimizer evaluation function.

While performance is not as good as the best combined approach of other investigators, it is as good as the best of the individual methods. With more accurate NER, as well as post-filtering using knowledge contained in online databases, the system may perform even better. Data sets and gold-standard files used in this work are available for download at [30].

## Methods

In this section we present our gene and protein synonym extraction algorithm, and our evaluation methods.

### Algorithm

We approached the problem of gene and protein name synonym extraction as a problem in mathematical network analysis. In the network, nodes are gene and protein names and symbols, and edges are labeled with the number of times the connected names have occurred in a text source together (i.e., the co-occurrence count). An initial set of synonym pair "seeds" is used to search through the text corpus for text patterns in which those synonym pairs occur. Occurrences of gene and protein names are replaced with a regular expression that matches a wide variety of possible gene and protein names. This regular expression is designed to have high recall for single word gene and protein names and symbols, at the expense of low precision. Then these patterns are matched against the corpus, extracting text patterns that include co-occurrences between pairs of names that are potential synonyms. The name co-occurrences extracted by the patterns are used to construct a gene name synonym network, and this network is mathematically analyzed to determine the combination of patterns that produces the strongest set of synonyms. The new synonyms with the highest confidence are then used as seeds in the next iteration of the algorithm. This process can be repeated for a set number of iterations, or until no new high confidence synonym pairs are found.

The regular expression used to identify gene and protein names is very non-specific: **([^\s,/%<>;+&()=\[\]\?\$\'\"]{3,14})**. The pattern excludes some punctuation and other special characters, but allows letters, numbers, as well as the period and colon characters. Gene and protein names are required to be between 3 and 14 characters long. The system then applies a set of heuristic rules to further screen out non-gene names. The name must not be in a stop list of words and patterns found during system development to be confused with gene and protein names (e.g., "RNA", "DNA", ".com"). The name may not begin with a digit, dash, colon, period, or asterisk, and may not end with a dash, period, or colon. Furthermore, the name may not contain only lowercase characters. All uppercase, a mix of upper and lowercase characters, or a combination of letters and numbers is required. These rules favor recall over precision.

The synonym text patterns are extracted from the text surrounding a pair identified synonyms. The system requires the synonym pairs to be within 4 words of each other, and includes zero or one words to either side of the synonym pair. For example, if **(CIP1, WAF1) **is an initial seed pair, and the text corpus includes the sentence:

Two percent or greater nuclear staining with WAF1/CIP1 monoclonal antibody was determined by hazard ratio analysis to constitute positive p21 expression. [31]

Then the system will extract the following patterns, where **$GENE$ **stands for the gene and protein name matching regular expression:

• **$GENE$/$GENE$**

• **with $GENE$/$GENE$**

• **$GENE$/$GENE$ monoclonal**

• **with $GENE$/$GENE$ monoclonal**

These patterns can then be applied to the text corpus to find name co-occurrences. For example, using the pattern **with $GENE$/$GENE$**, the system will extract the co-occurrence pairs **(CARD15, NOD2) **and **(MMAC, PTEN) **from the following sentence fragments found in the corpus respectively:

Of the children with NOD2/CARD15 variants...[32]

Human glioma xenografts treated with PTEN/MMAC gene transfer exhibited...[33]

**Figure 7:**
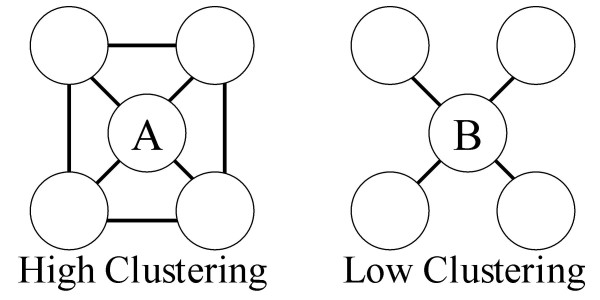
Representation of high and low clustering co-occurrence networks.

Given a set of patterns and the set of co-occurrences found by each pattern, the algorithm selects the best combination of patterns by evaluating the structure of the network created by the co-occurrences. The metric used to compare network structures is based on clustering coefficient measures [34]. A pattern is required to occur in the text a minimum of four times. The assumption is made that good synonym co-occurrence networks will have many separate, internally tightly linked clusters, since synonyms of synonyms should also have co-occurrences in the network. Figure 7 pictorially shows high versus low clustering co-occurrence networks.

The quality of a co-occurrence network is taken to be the ratio of the mean clustering coefficient (MCC) over the mean non-clustering coefficient (MNCC), and is computed as:





*quality *= *MCC */ *MNCC *    (3)

where *C *is the number of nodes in the network, *n(c) *is the list of neighbors for node *c*, *w(a,b) *is the number of co-occurrences seen between *a *and *b*, and *cmb(m, n) *is the standard combination function giving the number of combinations of *m *items taken *n *at a time. The minimum quality score is zero, the maximum is open ended and depends upon the number of nodes in the network and how interconnected they are. It is possible that a simpler measure could also work, however using MCC alone was considered but rejected because it favors larger lightly connected networks over smaller highly connected networks. The MNCC takes into account the size of the network and the number of nodes not connected to a given node. Note that MCC is only defined for nodes with two or more neighbors. A simpler measure of summing the weights for all the shared neighbors was considered, but not implemented. Analytically, it appears to give too much weight to a single very common synonym pair that is falsely connected to the node being measured. Computing the MCC/MNCC of the node pair averages the inter-connectivity across all the nodes connected to a pair of nodes, and therefore should be more accurate for groups of pairs synonymous to each other.

Finding the set of patterns which produce the network with the highest quality measure is a combinatorial optimization problem; the co-occurrences found by each pattern can either be included in the network or not. One of the best methods of solving this type of problem uses a genetic algorithm to optimize the combination of patterns chosen. We have chosen a variation of the canonical genetic algorithm that uses rank-order-based selection pressure [35,36]. It is used simply as a combinatorial optimizer. This variation was chosen because it works well and is easy to implement. Other genetic algorithm variants likely would perform just as well.

Once the set of patterns and their associated co-occurrences are chosen, the algorithm extracts synonym pairs from the co-occurrence network. This is done using a graph traversal algorithm much like Dijkstra's shortest path algorithm [37], and extracts synonym pairs explicitly found in the text as well as those that can be inferred by following the synonym relationships represented by the edges in the network. For example, if A is a synonym of B, and B is a synonym of C, then A is likely a synonym of C. During system training it was found to be best to restrict inference to network edges that had co-occurrence counts of 2 or greater.

In order to proceed with another iteration of the algorithm, the best synonym pairs must be chosen to use as seeds in the next iteration. Confidence in individual synonym pairs is determined using two network-based metrics. First, the overall confidence in a synonym pair with a given co-occurrence count *n *is estimated by computing the probability of seeing less than that occurrence count in a random graph with the same number of nodes and edges. This is computed as:



where *M *is the total number of co-occurrences, *N *is the number of nodes in the network, and *μ *= *N/M*. During training it was found that a confidence threshold of 0.999 gave the best results.

Synonym pairs with confidence greater then the threshold are then ordered by another network-based metric that measures the local clustering for the pair of nodes representing the synonym pair. The individual node clustering (CC) non-clustering (NCC) coefficients are computed, resulting in a local clustering metric (LCM) for each synonym pair:







Patterns are then extracted from the text using the high confidence synonym pairs as seeds, choosing the highest local clusterings first. The number of patterns to evaluate at each iteration was limited to 150, which was found to balance the quality of the results with the need to make the combinatorial optimization step solvable in a reasonable amount of time.

**Figure 8:**
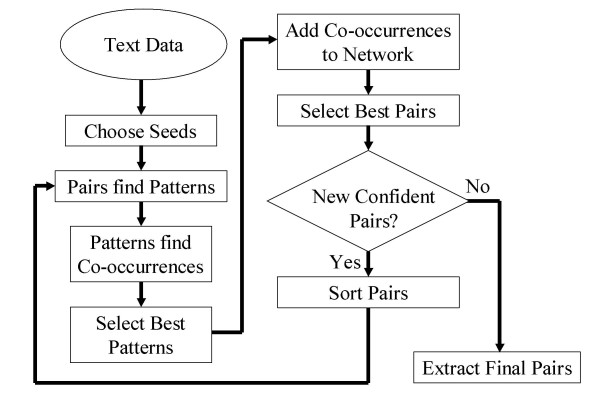
Synonym extraction algorithm.

Figure 8 illustrates the overall algorithm. The iterative pattern matching part of the algorithm is, like *Snowball*, based on the DIPRE approach of Brin. The novel parts of the algorithm presented here include the use of network-based metrics for evaluating the quality of patterns and synonym pairs, the use of a genetic optimization algorithm to determine the optimal set of patterns to use in extracting gene name synonyms, and the use of graph-based inference to infer synonym pairs not found explicitly in the text corpus.

### Experimental design

The experiment was performed in three steps. The first step was to develop and refine the algorithm detailed in the previous section on the training and validation data sets. Next, the algorithm was run on the MEDLINE records in the test set. Lastly, the quality of the extracted synonym list was evaluated by validating the synonymy of each extracted pair against a gold standard and then computing performance metrics.

#### Data sets

The training, validation, and test data sets used in this experiment consist of sentences extracted from approximately 50,000 abstracts from a year's worth of MEDLINE records containing the word "gene" for each set. Abstracts from 2001, 2002, and 2003 served as training, validation, and test sets, respectively. After downloading the MEDLINE records from PubMed, the records were parsed to extract the abstract field. The abstracts were then separated into sentences using a simple, lexically based sentence boundary detection algorithm. Finally, the sentences were screened to remove non-contributing sentences. These were sentences that did not contain at least two words that matched the gene and protein name regular expression discussed previously. This resulted in the three data sets used in this experiment each consisting of about 145,000 sentences each.

The training set was used for system development, debugging, and parameter tuning, as well as for choosing the initial set of seed synonym pairs. The validation set was used to verify the system and ensure that the chosen parameters worked as expected on multiple data sets. The test set was used to produce the experimental results.

#### Gold standards

Calculation of performance metrics for the experiment required gold standards for both precision and recall. Gene name synonyms available in on-line genomics databases served as the basis for both gold standards.

The approach used to create the precision gold standard consisted of downloading several genomics databases available on-line, extracting out the name, alias, and synonym fields, and combining them into a single gold standard for use in the computation of precision. The database snapshots that we used to construct our gold standard consisted of: SWISSPROT downloaded on 12/10/2003, FlyBase, Genew, and LocusLink, downloaded on 1/12/2004, and the MGI, and SGD databases downloaded on 1/22/2004.

The online databases do not contain all synonyms in common use. Orthographic variations (e.g., "WAF1" and "WAF-1") are often missing. Therefore during training and validation extracted candidate synonym pairs that were not found in the precision gold standard were manually reviewed for pairs that were likely to be correct (e.g. "CONNEXIN32" and "CX-32"), and these pairs were checked by reviewing MEDLINE for supporting information in the titles and abstracts. Manually verified pairs were added to the precision gold standard for use in scoring the results from the test data.

Creation of the recall gold standard was more challenging. Typically an accurate gold standard requires multiple experts to agree on definitions and then manually review the literature for the information in question, comparing multiple expert opinions and computing inter- and intra-rater agreement. Considering the large amount of text used, the expert resources were not available to use this method. Instead a simpler method was employed, based on the approach used by Yu and Agichtein.

To approximate a recall gold standard, all of the synonym pairs extracted from the SWISSPROT database [38] were compared to all of the sentences in the test collection. If both symbols of a synonym pair given in SWISSPROT were present together in at least one sentence in the test collection, that synonym pair was included in the recall gold standard. This resulted in a recall gold standard set of 483 synonym pairs for the test collection. While this may bias the recall gold standard towards the gene and protein names present in SWISSPROT, the bias is independent of any feature of the algorithm. Additionally, using a recall gold standard construction method like that of Yu and Agichtein facilitates later comparison of results.

Note that even though a pair of gene synonymous names from SWISSPROT may be present in a single sentence of the test set, it may be impossible for this or any other pattern-based algorithm to extract the pair. The synonyms could be separated by too many words, or the synonym pair may not occur in a repeated pattern. Nevertheless, a recall gold standard constructed by this method provides a useful benchmark.

## Abbreviations

clustering coefficient (CC)

local clustering metric (LCM)

mean clustering coefficient (MCC)

mean non-clustering coefficient (MNCC)

non-clustering coefficient (NCC)

named entity recognition (NER)

## Authors' contributions

AC wrote the software, ran the experiments, performed the data analysis, and drafted the manuscript. WH helped to design the study, participated in its coordination, and assisted in drafting the manuscript. CS provided support on gene nomenclature and databases. KS participated in the design of the algorithms and the evaluation methodology. All authors read and approved the final manuscript.
